# Upregulation of GLT-1 Expression Attenuates Neuronal Apoptosis and Cognitive Dysfunction via Inhibiting the CB1-CREB Signaling Pathway in Mice with Traumatic Brain Injury

**DOI:** 10.3390/biom15101408

**Published:** 2025-10-02

**Authors:** Bin Bu, Ruiyao Ma, Chengyu Wang, Shukun Jiang, Xiaoming Xu

**Affiliations:** 1 Department of Forensic Clinical Medicine, School of Forensic Medicine, China Medical University, Shenyang 110122, China; 2 Key Laboratory of Forensic Bio-Evidence Science, Shenyang 110122, China; 3 China Medical University Center of Forensic Investigation, Shenyang 110122, China; 4 Anshan Public Security Bureau, Anshan 114031, China; 5 First Clinical College, China Medical University, Shenyang 110001, China

**Keywords:** TBI, 2-AG, GLT-1, CREB, astrocyte

## Abstract

Background: Glutamate transporter 1 (GLT-1) plays a vital role in maintaining glutamate homeostasis in the body. A decreased GLT-1 expression in astrocytes can heighten neuronal sensitivity to glutamate excitotoxicity after traumatic brain injury (TBI). Despite its significance, the mechanisms behind the reduced expression of GLT-1 following TBI remain poorly understood. After TBI, the endocannabinoid 2-arachidonoyl glycerol (2-AG) is elevated several times. 2-AG is known to inhibit key positive transcriptional regulators of GLT-1. This study aims to investigate the role of 2-AG in regulating GLT-1 expression and to uncover the underlying mechanisms involved. Methods: A controlled cortical impact (CCI) model was used to establish a TBI model in C57BL/6J mice. The CB1 receptor antagonist (referred to as AM281) and the monoacylglycerol lipase (MAGL) inhibitor (referred to as JZL184) were administered to investigate the role and mechanism of 2-AG in regulating GLT-1 expression following TBI. Behavioral tests were conducted to assess neurological functions, including the open field, Y-maze, and novel object recognition tests. Apoptotic cells were identified using the TUNEL assay, while Western blot analysis and immunofluorescence were employed to determine protein expression levels. Results: The expression of GLT-1 in the contused cortex and hippocampus following TBI showed an initial decrease, followed by a gradual recovery. It began to decrease within half an hour, reached its lowest level at 2 h, and then gradually increased, returning to normal levels by 7 days. The administration of AM281 alleviated neuronal death, improved cognitive function, and reversed the reduction of GLT-1 caused by TBI in vivo. Furthermore, 2-AG decreased GLT-1 expression in astrocytes through the CB1-CREB signaling pathway. Mechanistically, 2-AG activated CB1, which inhibited CREB phosphorylation in astrocytes. This decreased GLT-1 levels and ultimately increased neuronal sensitivity to glutamate excitotoxicity. Conclusions: Our research demonstrated that the upregulation of GLT-1 expression effectively mitigated neuronal apoptosis and cognitive dysfunction by inhibiting the CB1-CREB signaling pathway. This finding may offer a promising therapeutic strategy for TBI.

## 1. Background

Traumatic brain injury (TBI) is a significant global public health and socioeconomic issue characterized by high rates of mortality and disability. TBI is categorized into two types: primary and secondary brain injuries, with secondary injuries being the primary focus for TBI treatment. Secondary injuries result from acute inflammatory responses that occur following primary brain injuries. These responses include ischemic injury, oxidative stress, excitotoxicity, and imbalances in endogenous regulatory mechanisms [1]. Among these factors, glutamate-mediated excitotoxicity plays a critical role in the progression of TBI. An imbalance in glutamate homeostasis can lead to neuronal cell apoptosis and death [2], exacerbate brain tissue damage, and result in various physical, emotional, cognitive, and other neurological dysfunctions [3]. Consequently, targeting glutamate-mediated excitotoxicity has emerged as a significant strategy in the therapeutic management of TBI.

Glutamate is the primary excitatory neurotransmitter in the central nervous system, responsible for transmitting rapid signals and playing a key role in learning, memory, and synaptic plasticity [4]. In a healthy brain, glutamate-mediated transmission occurs at the tripartite synapse, which consists of the presynaptic membrane, the postsynaptic membrane, and astrocytes, and glutamate release and clearance are tightly regulated [5]. Glutamate released from synaptic vesicles interacts with ionotropic and metabotropic receptors on presynaptic and postsynaptic membranes and astrocytes [6]. Excess glutamate is cleared from the extracellular space by excitatory amino acid transporters (EAATs) found in neurons and astrocytes [7]. Currently, five EAATs have been identified. EAAT1, also known as the glutamate and aspartate transporter (GLAST), and EAAT2, referred to as glutamate transporter 1 (GLT-1), are predominantly expressed in astrocytes [8,9]. EAAT4 and EAAT5 are primarily found in neurons. Among these, GLT-1 is the most widely distributed in the central nervous system, responsible for approximately 90% of synaptic glutamate clearance in astrocytes [10]. Therefore, any dysregulation of GLT-1 expression and function in astrocytes may significantly contribute to excitotoxicity.

Several studies have shown that GTL-1 levels decreased rapidly and were widely reduced throughout the brain following TBI [11,12,13]. However, the underlying mechanisms remain unclear. Understanding how GLT-1 expression is reduced may provide promising molecular targets for increasing GLT-1 levels and reversing glutamate excitotoxicity caused by TBI. Piao et al. found that an increase in brain thrombin levels following TBI, which induced a decrease in GLT-1 levels in hippocampal astrocytes via activation of the PAR-1 receptor [3]. A recent study has shown that TBI reduced GLT-1 and increased adenosine 2A receptor (A_2A_R) levels in the hippocampus, and GLT-1 levels were restored in mice treated with istradefylline (A_2A_R inhibitor)/ceftriaxone combination [14]. There is growing evidence that both nuclear factor kappa-light-chain-enhancer of activated B cells (NF-κB) and cAMP response element-binding protein (CREB) are essential positive transcriptional regulators that increase GLT-1 expression by binding to DNA-binding sites in the GLT-1 promoters [15]. Additionally, various stimulants activate NF-κB and CREB, leading to heightened GLT-1 expression through different signaling proteins. Extracellular signal-regulated kinase (ERK), mammalian target of rapamycin (mTOR), and Akt have been shown to modulate NF-κB activation to regulate GLT-1 expression [16,17]. Furthermore, the inhibition of protein kinase A (PKA), an upstream activator of CREB, blocks the effect of 17β-estradiol and tamoxifen in promoting GLT-1 expression [18].

The endocannabinoid system (ECS) consists of endocannabinoids, cannabinoid receptors, and enzymes responsible for the synthesis and degradation of endocannabinoids. It plays a role in the pathological processes that occur following TBI. After TBI, there is an increase in the synthesis and release of endocannabinoids. Notably, 2-arachidonoyl glycerol (2-AG), the most abundant endogenous cannabinoid, can rise to more than ten times its normal level within a short period [19]. The endocannabinoids, which are released from the postsynaptic neuron, inhibit retrogradely the release of neurotransmitters from presynaptic neurons. They also regulate various functions of astrocytes by increasing cytosolic Ca^2+^ signals through CB1 receptor activation in these cells. Notably, 2-AG has significant inhibitory effects on NF-κB and CREB, which are important positive transcriptional regulators of GLT-1 [20,21,22]. Furthermore, recent studies have demonstrated that astrocytes express proteins related to the ECS [23], suggesting that 2-AG may play a role in regulating GLT-1 expression in astrocytes.

In summary, GLT-1, a critical transporter of glutamate, is significantly reduced following TBI. However, the underlying mechanisms for this decrease are not yet fully understood. Notably, following TBI, levels of 2-AG, the primary endocannabinoid, as well as the inhibition of essential positive transcriptional regulators of GLT-1, exhibit a significant increase [19], suggesting that 2-AG may play a role in regulating GLT-1 expression. Therefore, this study aims to clarify the regulatory effect of 2-AG on GLT-1 expression after TBI, as well as the potential molecular signaling pathways involved. In addition, we use behavioral methods to observe whether regulating the expression of GLT-1 can improve neurological dysfunction caused by TBI. Such insights may provide valuable targets for therapeutic intervention in cases of TBI.

## 2. Materials and Methods

### 2.1. Animals

All experimental procedures were approved by the Ethics Committee on Animals of China Medical University (CMU2021548). A total of 219 wild-type C57BL/6 male mice (10 weeks old, Laboratory Animal Centre of China Medical University) were randomly assigned to a TBI or sham group. Mice were housed in a controlled environment (20–22 °C; 12-h light: dark on a reversed light cycle) for 1 week before the experiments. Mice had unlimited access to food and water in their home cages, and all efforts were made to minimize the number of animals studied.

### 2.2. Controlled Cortical Impact (CCI) Model

CCI model was utilized to induce a moderate TBI model as described in previous studies [24,25]. Briefly, mice were placed on a stereotaxic apparatus after being anesthetized with 1.4% isoflurane. A midline scalp incision was made to expose the skull. A 4 mm craniotomy was performed 2.5 mm posterior to the bregma and 2.5 mm to the right of midline. Using a craniocerebral strike apparatus (PinPoint™ PCI3000, Hatteras Instruments, Grantsboro, NC, USA), a vertical impact was delivered to the cortex with a 3 mm diameter impactor at a velocity of 1.5 m/s for a residence time of 50 ms and a depth of 1 mm. The scalp was then sutured. The comatose mice were placed on a heating pad set to 37 °C and returned to their cages once their vital signs stabilized. Mice in the sham group underwent the same surgical procedure but without any cortical impact.

### 2.3. Behavior Test

The behavioral test started at 7 pm 1–15 days after TBI, with the surrounding envi-ronment kept quiet and dark.

#### 2.3.1. Open Field Test

The open field test was used to detect locomotor activity and anxiety-like behavior as described in a previous study [26] with modifications. Briefly, the movement of the test mouse was measured with a camera in the peripheral and central zones of a 40 cm × 40 cm × 30 cm plastic box. The mouse was acclimated in the box for 5 min before the experiment. The distance and trajectory of the mouse’s movement were recorded for 10 min using the SMART2.5.21 system (San Diego Instruments, San Diego, CA, USA). The entire area of the box was cleaned and wiped with 75% alcohol before proceeding to the next test.

#### 2.3.2. Novel Object Recognition Test

The novel object recognition test was conducted to assess learning and memory in mice as described in a previous study [27]. Briefly, the mouse was first placed in the middle of an empty box and allowed to explore freely for 10 min. Then, two identical objects were placed in opposite quadrants of the box, and the mice were introduced gently to explore the objects for 5 min. After this exploration period, the mice were returned to their home cage. After 10 min, one of the objects was replaced with a novel object that differed in color and shape. The cumulative time spent exploring each object was then recorded for an additional 5 min by using the SMART2.5.21 system. The box and objects were thoroughly cleaned with 75% ethanol after each trial. The Recognition Index (RI) was calculated as the percentage of time spent exploring the novel object relative to the total time spent exploring both objects.

#### 2.3.3. Y-Maze Test

The Y maze was used to test the cognitive function in mice as described in a previous study [28] with modifications. The Y maze test was performed in a Y-shaped maze, which consisted of three arms (10 cm × 30 cm ×20 cm, width × length × height), with a 120° angle from each other. Three arms were randomly designated as the start arm, the novel arm, and another arm. The Y-maze test included two trials separated by a 2-h interval. In the first trial (training), the novel arm was blocked, and the mouse was placed in the center of the maze and allowed to explore the start arm and another arm for 10 min. In the second trial (retention), all three arms were opened, and the mouse was allowed to move freely for 5 min from the end of the same start arm. The number of arm entries and the time spent in each arm were recorded using the SMART2.5.21 system. Maze arms were cleaned with 75% ethanol solution between trials.

### 2.4. Drug Administration

AM281 (CB1 receptor antagonist, APExBIO, Houston, TX, USA) and JZL184 (the monoacylglycerol lipase (MAGL) inhibitor, APExBIO, Houston, TX, USA) were prepared and dissolved in a vehicle containing Tween 80, dimethyl sulfoxide, and saline (1:1:18, *v*/*v*).

Studies have demonstrated that AM281 significantly blocked the effect of 2-AG through CB1 receptor, while JZL184 significantly increased the concentration of 2-AG in the brain by inhibiting MAGL (2-AG degradation key enzyme) [28,29]. Mice were injected intraperitoneally with 3 mg/kg of AM281 [29], 16 mg/kg JZL184 [29] or vehicle 30 min post-TBI, respectively, and then once a day for 7 consecutive days (total 8 injections).

### 2.5. Brain Tissue Harvest and Protein Level Quantification

The mice were killed by decapitation (for Western blot analysis). Approximately 4 mm of cerebral cortex around the injury area and the ipsilateral-injured hippocampus were harvested separately. The harvested brain cortex and hippocampus were homogenized on ice using immunoprecipitation buffer (10 mM Tris-HCl, pH 7.4, 150 mM NaCl, 2 mM EDTA, 0.5% Nonidet P-40) plus protease inhibitors (1 μg/mL aprotinin, 1 μg/mL leupeptin, 1 μg/mL pepstatin A). The lysates were collected and centrifuged at 12,000× *g* for 10 min, and protein concentration was measured using a BCA kit (P0012; Beyotime Biotechnology, Shanghai, China).

### 2.6. Western Blot

Western blotting quantification was performed as previously described [30]. Briefly, relative band density was quantified using ImageJ software (National Institutes of Health) and normalized to the total amount of protein (20 μg) loaded in each well as determined by β-actin. The Western blot images presented in the manuscript are just for representative purposes. Antibody information is presented in Supplementary Table S1.

### 2.7. Immunofluorescence

Mice were anesthetized with pentobarbital sodium (80 mg/kg), followed by heart perfusion with cold phosphate-buffered saline and 4% paraformaldehyde sequentially. The whole brain was harvested. Frozen sections (20 μm) were used for the immunofluorescence (IF) staining. IF staining was performed as previously described in the same experimental conditions [25]. The peripheral area of cortical injury and the CA1, CA3, and DG subregions of the hippocampus were listed as regions of interest and 9 slices from 3 mice per group were selected. Images were acquired using a laser scanning confocal microscope (TCS SP8; Leica) and the average fluorescence intensity of the 1000 × 1000 pixel area in the images was analyzed using ImageJ 1.8.0 software (National Institutes of Health) at the same setting. The antibodies used in this study are listed in Supplementary Table S1.

### 2.8. TUNEL Staining

A TUNEL BrightGreen Apoptosis Detection Kit (A111-01; Vazymem, Nanjing, China) was used as directed by the manufacturer. Briefly, the frozen sections were treated with proteinase K. Afterward, the tunnel reaction solution was added, and DAPI was used to stain the nuclei. Finally, apoptotic neuronal cells were visualized using a fluorescence microscope (Zeiss Axio Scan.Z1, Oberkochen, Baden-Württemberg, Germany). 9 slices from 3 mice per group were selected and the number of apoptotic neuronal cells in the peripheral area of cortical injury and the CA1, CA3, and DG subregions of the hippocampus was counted manually within a 1000 × 1000 pixel area in the images.

### 2.9. Statistical Analysis

All data are expressed as means ± SEM. Statistical analysis of differences in outcome measures was determined by *t*-test or one-way ANOVA using GraphPad Prism 9 statistical software (Graphpad Software Inc., La Jolla, CA, USA). One-way ANOVA without repeated measures for multiple comparisons was used, followed by Tukey’s test when F ratios were significant. Significant differences between the two groups were determined using the Student’s *t*-test. *p* values less than 0.05 were considered statistically significant.

## 3. Results

### 3.1. The Downregulation of GLT-1 Expression in Astrocytes Resulting from TBI Was Effectively Reversed by Inhibiting the CB1 Receptor

**Figure 1:**
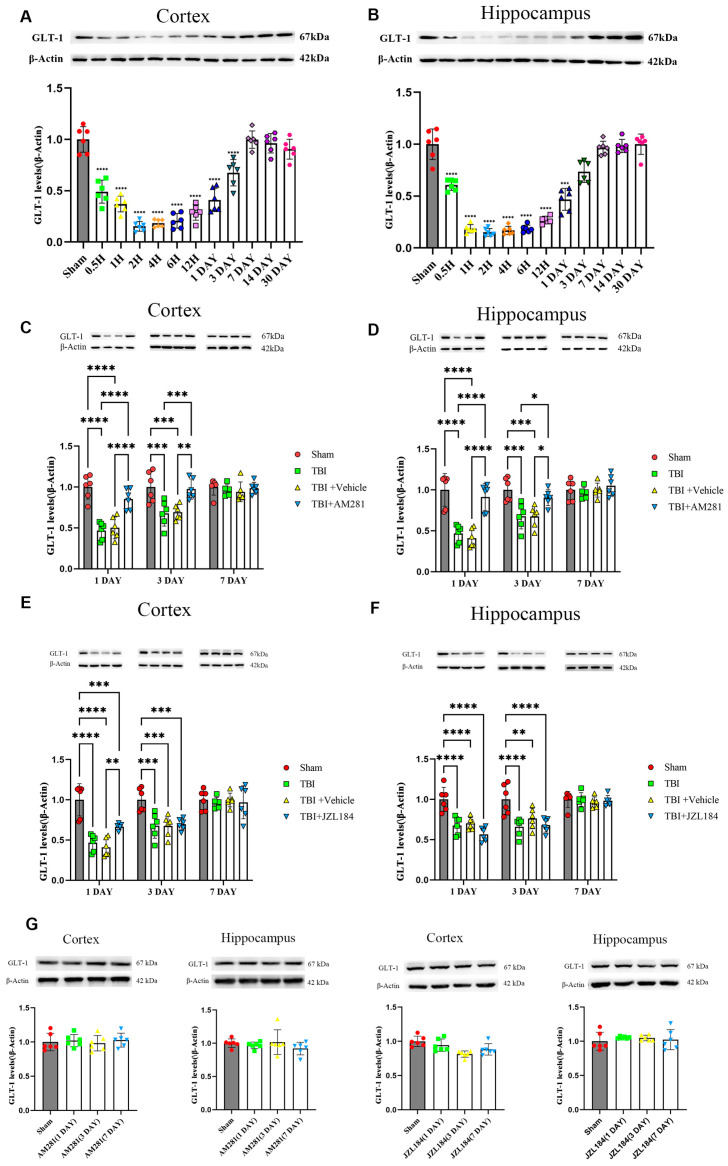
The decrease in GLT-1 expression after TBI was reversed by injecting AM281 to inhibit CB1 receptors, rather than by injecting JZL184 to inhibit the degradation of 2-AG. (**A**,**B**): Western blotting analysis of GLT-1 in the cortex (**A**) and hippocampus (**B**) of TBI mice at different time points post-TBI. (**C**–**F**): Western blotting analysis of GLT-1 in the cortex (**C**,**E**) and hippocampus (**D**,**F**) of TBI, Sham, TBI + AM281, and TBI + Vehicle group on days 1, 3, 7 post-TBI. (**G**): Western blotting analysis of GLT-1 in the cortex and hippocampus of Sham group on days 1, 3, 7 post-injection. *n* = 6 mice per group. All data were presented with the mean ± SEM. * *p* < 0.05, ** *p* < 0.01, *** *p* < 0.001, **** *p* < 0.0001.

The GLT-1 levels in the mouse brain were assessed through western blot analysis at various time intervals following TBI. The findings indicated a distinct pattern characterized by an initial decrease in GLT-1 levels, which was subsequently followed by a gradual recovery in both the contused cortex and hippocampus after TBI. As illustrated in Figure 1A,B, GLT-1 levels began to decline within thirty minutes, reaching their lowest point with significant reductions of 85.63% in the cortex and 85.13% in the hippocampus compared to the sham group at 2 h. GLT-1 levels gradually rose after this dip, returning to baseline levels 7 days post-TBI.

To determine whether ECS regulated GLT-1 expression, mice were intraperitoneally injected with the CB1 receptor antagonist AM281 and 2-AG degradation enzyme inhibitor JZL184. The cortex and hippocampus were collected and the GLT-1 levels were assessed using western blotting and immunofluorescence staining on days 1, 3, and 7 post-injection. The western blot results indicated a significant increase in GLT-1 expression in the cortex (Figure 1C) and hippocampus (Figure 1D) of mice injected with AM281 at 1 and 3 days, compared to the TBI group, with no significant difference compared to the sham group. Surprisingly, the injection of JZL184 did not exacerbate the decrease in GLT-1 expression (Figure 1E,F). By day 7, GLT-1 expression had returned to normal levels in both the cortex and hippocampus, and no significant differences were observed among the four groups.

**Figure 2:**
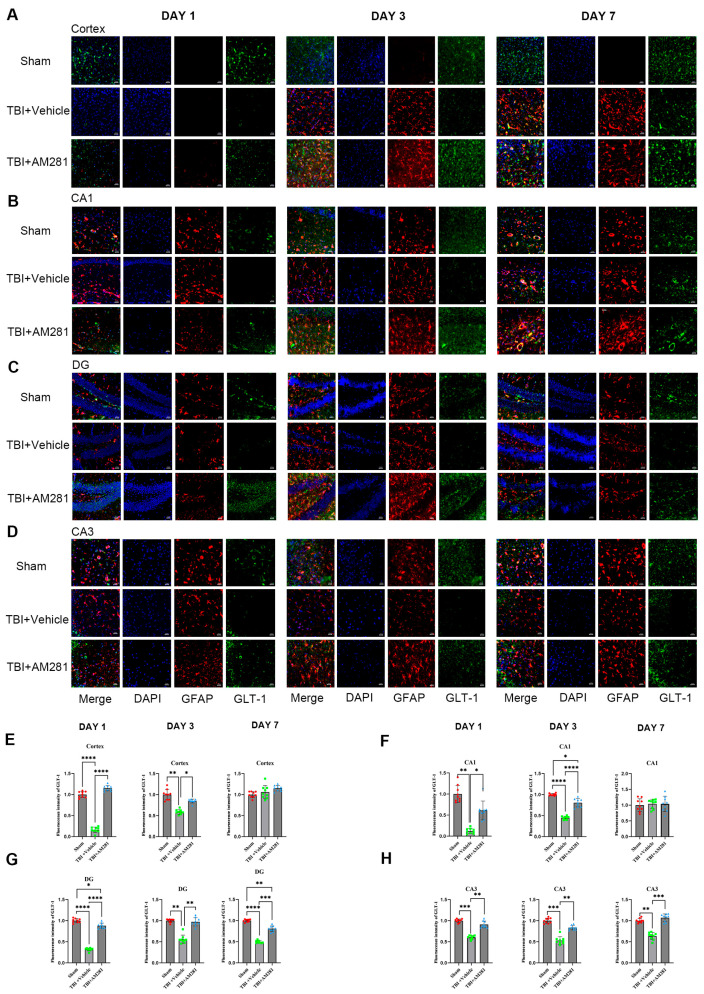
TBI-induced downregulation of GLT-1 expression in astrocytes was reversed by inhibiting the CB1 receptor. (**A**–**D**): Representative immunofluorescent images demonstrating the expression of GFAP (Red), DAPI (Blue), and GLT-1 (Green) in the cortex (**A**), CA1 (**B**), DG (**C**), and CA3 (**D**) of Sham, TBI + AM281, and TBI + Vehicle group on days 1, 3, 7 post-TBI. (**E**–**H**): Quantification of immunofluorescence images in (**A**–**D**). *n* = 9 slices from 3 mice per group. Bar = 10 μm. All data were presented with the mean ± SEM. * *p* < 0.05, ** *p* < 0.01, *** *p* < 0.001, **** *p* < 0.0001.

The expression of GLT-1 in astrocytes was detected through immunofluorescence staining. As indicated by Figure 2 and Supplementary Figure S1, following TBI, the GFAP levels increased in the peripheral cerebral cortex of the injured area on day 3 and 7. Notably, AM281 did not have an impact on this increase. Consistent with the results from western blot analysis, the downregulation of GLT-1 expression in astrocytes from the cortex, CA1, CA3, and DG subregions was reversed by inhibiting the CB1 receptor on days 1 and 3. This suggests that the CB1 receptor is involved in the regulation of GLT-1 expression. By day 7, GLT-1 expression had returned to normal levels in both the cortex and the CA1 subregions of the hippocampus. However, there remained a 36.58% reduction in the CA3 subregion and a 48.14% reduction in GLT-1 levels in the DG subregion compared to the sham group.

### 3.2. Reversing the Decrease in GLT-1 Expression Reduced Neuronal Apoptosis in the CA3 and DG Subregions of Mice with TBI

**Figure 3:**
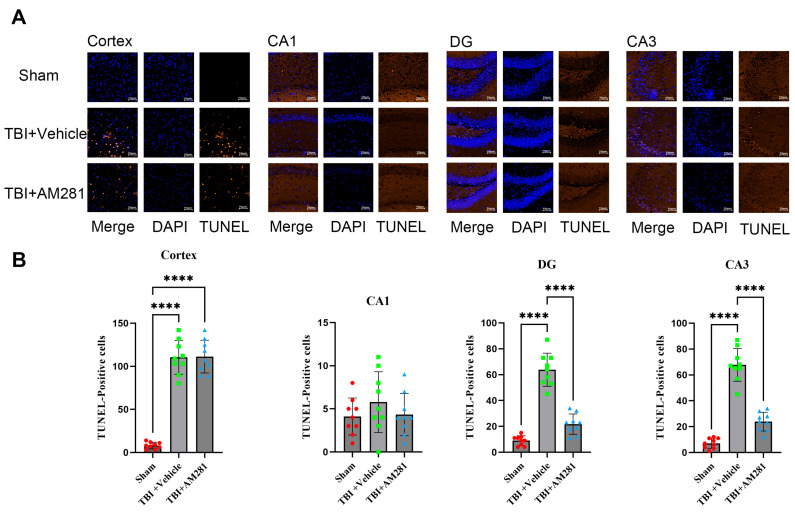
Inhibiting the CB1 receptor significantly mitigated neuronal apoptosis in the CA3 and DG subregions. (**A**): Representative immunofluorescent images demonstrating neuronal apoptosis (DAPI (Blue) and Tunnel (Brown)) in the cortex, CA1, DG, and CA3 of Sham, TBI + AM281, and TBI + Vehicle group on day 1 post-TBI. (**B**): Quantification of immunofluorescence images in (**A**). *n* = 9 slices from 3 mice per group. Bar = 10 μm. All data were presented with the mean ± SEM.**** *p* < 0.0001.

The investigation of neuronal apoptosis in the brains of mice was conducted using TUNEL staining. As shown in Figure 3, a significant increase in neuronal apoptosis was observed in the cortex, CA3, and DG subregions on day 1 following TBI, but no increase was observed in the CA1 subregion. Additionally, the administration of AM281 significantly mitigated neuronal apoptosis in the CA3 and DG subregions, although no effect was observed in the cortex.

### 3.3. GLT-1 Expression in the Hippocampus Was Significantly Increased by Inhibiting the CB1-CREB Signaling Pathway

**Figure 4:**
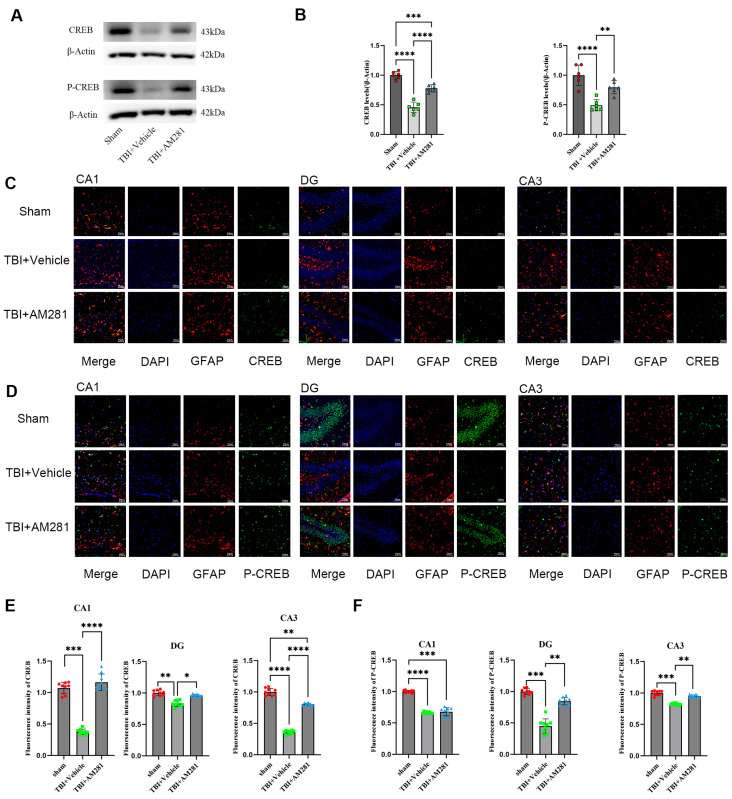
2-AG is involved in regulating GLT-1 expression in astrocytes via the CB1-CREB signal pathway. (**A**,**B**): Western blotting analysis of CREB and P-CREB in the hippocampus followed AM281 (*n* = 6 mice per group). (**C**,**D**): Representative immunofluorescent images demonstrating the expression of GFAP (Red), DAPI (Blue), and CREB (Green) (**C**) or P-CREB (Green) (**D**) in the CA1, DG, and CA3 of Sham, TBI + AM281, and TBI + Vehicle group on day 1 post-TBI (*n* = 9 slices from 3 mice per group). (**E**,**F**): Quantification of immunofluorescence images in (**C**,**D**). Bar = 10 μm. All data were presented with the mean ± SEM. * *p* < 0.05, ** *p* < 0.01, *** *p* < 0.001, **** *p* < 0.0001.

Given that CREB and NF-κB are the primary positive transcriptional regulators of GLT-1 expression, we initially examined the changes in the expression levels of both factors using western blot and immunofluorescence staining in vivo. We found that the expression of CREB and phosphorylated CREB (P-CREB) was significantly decreased in the hippocampus and in the astrocytes of the CA1, CA3, and DG subregions one day after TBI compared to the sham group. This decrease was reversed with an injection of AM281, although P-CREB levels in the CA1 subregion did not show improvement (Figure 4). Conversely, NF-κB (P65) expression was significantly increased; however, no changes were observed following AM281 treatment (Supplementary Figure S2). These findings suggest that 2-AG is involved in regulating GLT-1 expression through the CB1-CREB signaling pathway. Inhibiting this signaling pathway led to a significant upregulation of GLT-1 expression.

### 3.4. The Cognitive Impairment Resulting from TBI Was Improved by Inhibiting the CB1 Receptor

**Figure 5:**
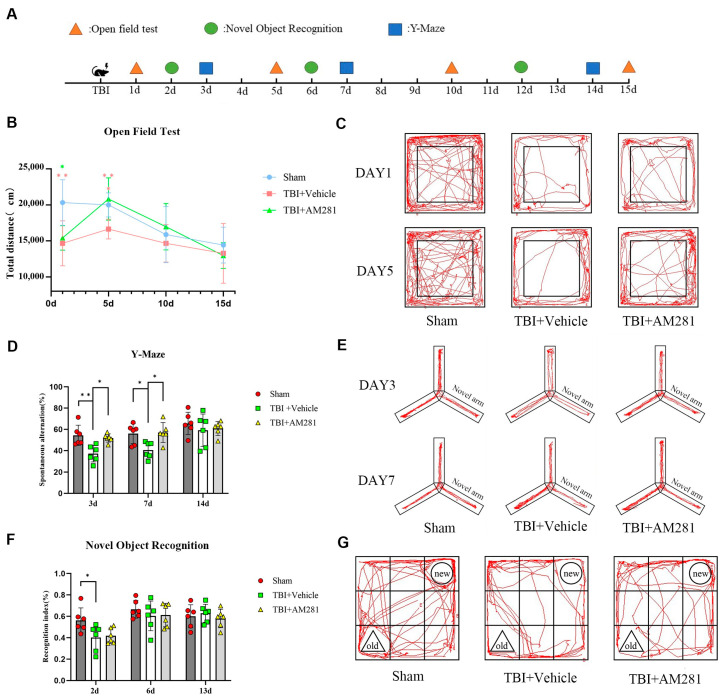
Blocking CB1 receptors improved cognitive impairment following TBI. (**A**): A schematic protocol for behavioral experiments. (**B**): Representative images showing the travel paths of Sham, TBI + Vehicle, and TBI + AM281 recorded in the open field test box. (**C**): Locomotor activity was assessed in the open field test as the total distance on days 1, 5, 10, and 15 post-TBI. (**D**): Representative images showing the travel paths of Sham, TBI + Vehicle, and TBI + AM281 were recorded in the Y-maze. (**E**): Spatial memory was evaluated in the Y-maze test as the spontaneous alternation on days 3, 7, and 14 post-TBI. (**F**): Representative images showing the travel paths of Sham, TBI + Vehicle, and TBI + AM281 were recorded in the novel object recognition test box. (**G**): Learning and memory were assessed in the novel object recognition test as the recognition index on days 2, 6, and 13 post-TBI. *n* = 6 mice per group. All data were presented with the mean ± SEM. * *p* < 0.05, ** *p* < 0.01.

The cognitive impairment resulting from TBI was evaluated using the open-field, Y-maze, and novel object recognition tests. In the open-field test (Figure 5C), we observed depressive-like behavior characterized by significantly reduced spontaneous and exploratory activity compared to the sham group on day 1 post-TBI. Treatment with AM281 improved TBI-induced depressive-like behavior by day 5. In the Y-maze test (Figure 5E), we identified learning and memory impairments in mice after TBI, as indicated by significantly lower spontaneous alternation ratios compared to the sham group at 3 days post-injury. This significant difference persisted until day 7. Treatment with AM281 improved the learning and memory deficits associated with TBI on day 3. In the novel object recognition test (Figure 5G), we found evidence of TBI-induced learning and memory impairment at 2 days, marked by a significantly reduced recognition index. However, there was no significant difference in recognition index between the mice treated with AM281 and those receiving vehicle treatment.

## 4. Discussion

TBI has a high incidence and poses significant risks in daily life, leading to neurotoxicity that can trigger various post-traumatic neurodegenerative diseases [31]. TBI is associated with a decrease in glutamate transporters, specifically GLT-1, which plays a vital role in preventing excitotoxicity. However, the underlying mechanisms remain unclear. In this study, we demonstrate for the first time that the endocannabinoid 2-AG inhibits CREB phosphorylation in astrocytes through CB1 receptor. This inhibition ultimately results in a reduction of GLT-1 levels in mice following TBI.

GLT-1 is the most widely distributed glutamate transporter in the central nervous system, accounting for approximately 90% of synaptic glutamate clearance in astrocytes [10]. Goodrich et al. demonstrated that GLT-1 expression was reduced in the cortex following TBI in rats, with a notable decrease observed in the ipsilesional cortex compared to the contralesional cortex [11]. Similarly, Gupta et al. investigated the downregulation of GLT-1 expression in the pericontusional cortex of old male mice after TBI. They found a significant decrease in GLT-1 expression at both the transcript and protein levels in older TBI mice compared to adult TBI mice [12]. Our study found that GLT-1 levels decreased rapidly and were widespread throughout almost the entire hemisphere ipsilateral to the injury following TBI. GLT-1 expression exhibited a consistent pattern of initial decline followed by a gradual recovery in the contused cortex and hippocampus after TBI. Specifically, GLT-1 levels began to decrease within half an hour, reached their lowest point at 2 h, and then gradually increased, returning to normal levels by day 7. However, the underlying mechanism is still unclear, especially the rapid decline in a short period of time, which may be the internalization of transporters triggered by an increase in extracellular glutamate following TBI. Like many other membrane transporters, the trafficking of GLT-1 protein to and from the plasma membrane provided a means of rapidly regulating its activity. A study showed that an increase in extracellular glutamate reduced cell surface GLT-1 in a dose-dependent manner, which promoted the association with GLT-1 of the bridging protein β-arrestin and the ubiquitin ligase Nedd4-2, leading to increased ubiquitination of GLT-1, followed by endocytosis and lysosomal degradation [32]. We also observed changes in GLT-1 levels in the CA1, CA3, and DG subregions of the hippocampus, which are crucial for memory and cognition. By day 7, GLT-1 expression had returned to normal levels in the CA1 subregion. However, significant reductions in GLT-1 expression persisted in the CA3 and DG subregions in the TBI group compared to the sham group. Additionally, more severe neuronal apoptosis was observed in the CA3 and DG subregions on day 1 following TBI, but not in the CA1 subregion, suggesting that low GLT-1 expression has a greater impact on the apoptosis of CA3 and DG neurons. Zhang et al. found that the baseline GLT-1 expression in the CA3 and DG subregions was significantly higher than in the CA1 subregion, possibly contributing to delayed neuronal death in the CA3 and DG following cerebral ischemia. Their findings indicate that downregulation of GLT-1 expression and function can induce neuronal death [33]. Furthermore, Schumm et al. reported that the functionality of the CA3 subregion was more susceptible to impairment in a hippocampal network model of mild TBI [34]. Therefore, targeting the CA3 and DG subregions may represent an effective therapeutic strategy to mitigate neurodegeneration and improve cognitive impairments caused by TBI.

2-AG is the most abundant endogenous cannabinoid. Following TBI, its levels in the hippocampus of mice increase to more than six times the normal amount within one day and remain over three times the normal level five days post-injury [29]. Released from postsynaptic neurons, 2-AG not only inhibits the release of neurotransmitters from presynaptic neurons, but also regulates various functions of astrocytes through the activation of CB1 receptors. Interestingly, 2-AG has significant inhibitory effects on NF-κB and CREB, the crucial positive transcriptional regulators of GLT-1 [20,21,22]. This suggests that 2-AG may contribute to the downregulation of GLT-1 expression following TBI. To test this hypothesis, mice were injected intraperitoneally with a CB1 receptor antagonist AM281 after TBI. Surprisingly, the downregulation of GLT-1, CREB, and P-CREB expression in astrocytes caused by TBI was reversed by inhibiting the CB1 receptor one day after the injury, but no changes in NF-κB (P65) expression were observed. These findings indicate that 2-AG is involved in regulating GLT-1 expression via the CB1-CREB signaling pathway. However, the injection of JZL184 did not exacerbate the decrease in GLT-1 expression following TBI. It is speculated that the possible reason is that the significantly elevated 2-AG in the brain after TBI is sufficient for the activation of CB1 receptors on astrocytes, so JZL184 and TBI did not have a synergistic effect on the reduction of GLT-1 expression. In fact, we found that the expression level of GLT-1 in the cortex of mice injected with MAGL inhibitor JZL184 increased compared to the TBI group on day 1 (Figure 1E), while there was no significant difference at subsequent time points. We speculate that the elevated 2-AG may regulate GLT-1 expression through non-CB1 receptor-dependent signaling pathways, which requires further research in the future.

Our results indicate that mice with TBI display short-term memory impairment and depression-like behavior, accompanied by a significant amount of neuronal apoptosis in the CA3 and DG subregions of the hippocampus. Importantly, inhibiting the CB1 receptor through the injection of AM281 significantly reduced neuronal apoptosis and abnormal behaviors while enhancing GLT-1 expression. The DG and CA3 are critical structures involved in the editing and storing of memory within the hippocampus, each with distinct cellular structures and functions. The DG plays a vital role in forming hippocampal memories by receiving information from the entorhinal cortex and transmitting it to the CA3 region, which is responsible for encoding, storing, and retrieving memories [35]. Therefore, improving neuronal apoptosis in these specific hippocampal subregions can help mitigate memory and cognitive impairments resulting from TBI. Neuronal damage and necrosis following TBI are exacerbated by glutamate accumulation, which leads to excitotoxicity associated with downregulated GLT-1 expression. Consistent with our findings, several studies have demonstrated that increasing GLT-1 expression may provide neuroprotective effects in TBI models. For instance, Cui et al. investigated the neuroprotective effects of ceftriaxone in a rat TBI model and found that ceftriaxone treatment could upregulate GLT-1 expression and inhibit post-TBI neuronal autophagy [36]. Additionally, Sun et al. proposed a new mechanism for cognitive impairment involving astrocytic P-connexin 43, which promotes neuronal autophagy by downregulating GLT-1 expression in the hippocampus after TBI in rats [37]. Furthermore, Piao et al. provided evidence that TBI decreased astrocytic GLT-1, which correlates with increased extracellular glutamate and depressive-like behavior [3]. These findings suggest that alterations in GLT-1 expression play a crucial role in the pathophysiology of TBI, and interventions aimed at targeting GLT-1 expression may offer neuroprotective benefits in TBI models.

Previous studies provided evidence that inhibition of MAGL by injection of JZL184, which elevated 2-AG levels and enhanced 2-AG signaling, produced neuroprotective effects in TBI [38,39,40]. 2-AG accumulation derived from MAGL inhibition leads to the activation of cannabinoid receptors. Since CB1 receptors are mainly expressed in the central nervous system, the neuroprotective effects of MAGL inactivation are mediated by CB1 receptors [41]. Interestingly, the neuroprotective effects are also observed by inhibiting CB1 in our current study, which seems to be inconsistent with the above studies. However, there are reports indicating that the neuroprotective effects appear not to be guided through the cannabinoid receptor-dependent pathway, but by lowering pro-inflammatory eicosanoids. Attenuated neuroinflammatory responses in animal models of PD and AD were not reversed by cannabinoid receptor antagonists, indicating that the protective effects observed were mainly due to decreased levels of prostaglandins and cytokines in the brain [42,43]. Moreover, CB1 antagonists can not only attenuate the effects of CB1 agonists, but also act as inverse agonists, which can elicit responses opposite in direction from those elicited by CB1 agonists [44]. Studies show that blocking CB1 produces neuroprotective effects on retinal degeneration [45,46]. Those studies disclose the multiple connections of the ECS with other signaling pathways in the CNS. In addition, there are some limitations in our current research, such as the lack of positive and negative controls, and further validation with large samples is needed in the future.

## 5. Conclusions

Our current findings suggest a new mechanism by which TBI leads to the downregulation of astrocyte GLT-1 levels. Selectively inhibiting the CB1-CREB signaling pathway may reduce neuronal apoptosis and cognitive dysfunction, potentially providing a new therapeutic strategy for TBI. Further research is necessary to explore this avenue.

## Data Availability

The original contributions presented in this study are included in the article/supplementary material. Further inquiries can be directed to the corresponding author(s).

## Supplementary Materials

The following supporting information can be downloaded at: https://www.mdpi.com/article/10.3390/biom15101408/s1, Table S1: Antibodies used in the present study for WB and IF; Figure S1: TBI-induced upregulation of GFAP expression in astrocytes of cortex was not reversed by inhibiting the CB1 receptor; Figure S2: TBI-induced upregulation of P65 expression in astrocytes was not reversed by inhibiting the CB1 receptor.

## Abbreviations

2-AG	2-arachidonoyl glycerol
CB1	Cannabinoid type 1
CCI	Controlled cortical impact
CREB	Cyclic-AMP response binding protein
DMEM	Dulbecco’s modified Eagle’s medium
EAATs	Excitatory amino acid transporters
ECS	Endocannabinoid system
ERK	Extracellular signal-regulated kinase
FBS	Fetal bovine serum
GFAP	Glial fibrillary acidic protein
GLAST	Glutamate and aspartate transporter
GLT-1	Glutamate transporter-1
IF	Immunofluorescence
MAGL	Monoacyl glycerol lipase
mTOR	Mammalian target of rapamycin
NF-κB	Nuclear factor kappa-light-chain-enhancer of activated B cells
PKA	Protein kinase A
RI	Recognition Index
TBI	Traumatic brain injury
WB	Western blot

## References

[1] Zibara K., Ballout N., Mondello S., Karnib N., Ramadan N., Omais S., Nabbouh A., Caliz D., Clavijo A., Hu Z. (2019). Combination of drug and stem cells neurotherapy: Potential interventions in neurotrauma and traumatic brain injury. Neuropharmacology.

[2] Yi J.-H., Hazell A.S. (2006). Excitotoxic mechanisms and the role of astrocytic glutamate transporters in traumatic brain injury. Neurochem. Int..

[3] Piao C.-S., Holloway A.L., Hong-Routson S., Wainwright M.S. (2019). Depression following traumatic brain injury in mice is associated with down-regulation of hippocampal astrocyte glutamate transporters by thrombin. J. Cereb. Blood Flow Metab..

[4] Zhou Y., Danbolt N.C. (2014). Glutamate as a neurotransmitter in the healthy brain. J. Neural Transm..

[5] Perea G., Navarrete M., Araque A. (2009). Tripartite synapses: Astrocytes process and control synaptic information. Trends Neurosci..

[6] Bauminger H., Gaisler-Salomon I. (2022). Beyond NMDA Receptors: Homeostasis at the Glutamate Tripartite Synapse and Its Contributions to Cognitive Dysfunction in Schizophrenia. Int. J. Mol. Sci..

[7] Iovino L., Tremblay M., Civiero L. (2020). Glutamate-induced excitotoxicity in Parkinson’s disease: The role of glial cells. J. Pharmacol. Sci..

[8] Zhou Y., Danbolt N.C. (2013). GABA and Glutamate Transporters in Brain. Front. Endocrinol..

[9] Zhou Y., Hassel B., Eid T., Danbolt N.C. (2019). Axon-terminals expressing EAAT2 (GLT-1; Slc1a2) are common in the forebrain and not limited to the hippocampus. Neurochem. Int..

[10] Bélanger M., Magistretti P.J. (2009). The role of astroglia in neuroprotection. Dialog. Clin. Neurosci..

[11] Goodrich G.S., Kabakov A.Y., Hameed M.Q., Dhamne S.C., Rosenberg P.A., Rotenberg A. (2013). Ceftriaxone Treatment after Traumatic Brain Injury Restores Expression of the Glutamate Transporter, GLT-1, Reduces Regional Gliosis, and Reduces Post-Traumatic Seizures in the Rat. J. Neurotrauma.

[12] Gupta R.K., Prasad S. (2013). Early down regulation of the glial Kir4.1 and GLT-1 expression in pericontusional cortex of the old male mice subjected to traumatic brain injury. Biogerontology.

[13] Lim S.-W., Su H.-C., Nyam T.-T.E., Chio C.-C., Kuo J.-R., Wang C.-C. (2021). Ceftriaxone therapy attenuates brain trauma in rats by affecting glutamate transporters and neuroinflammation and not by its antibacterial effects. BMC Neurosci..

[14] Alves M., Gerbatin R., Kalmeijer R., Fedele D., Engel T., Boison D. (2025). Adenosine A2A receptor and glial glutamate transporter GLT-1 are synergistic targets to reduce brain hyperexcitability after traumatic brain injury in mice. Neuropharmacology.

[15] Pajarillo E., Rizor A., Lee J., Aschner M., Lee E. (2019). The role of astrocytic glutamate transporters GLT-1 and GLAST in neurological disorders: Potential targets for neurotherapeutics. Neuropharmacology.

[16] Karki P., Hong P., Johnson J., Pajarillo E., Son D.-S., Aschner M., Lee E.Y. (2018). Arundic Acid Increases Expression and Function of Astrocytic Glutamate Transporter EAAT1 Via the ERK, Akt, and NF-κB Pathways. Mol. Neurobiol..

[17] Abousaab A., Uzcategui N.L., Elsir B., Lang F. (2016). Up-Regulation of the Excitatory Amino Acid Transporters EAAT1 and EAAT2 by Mammalian Target of Rapamycin. Cell. Physiol. Biochem..

[18] Karki P., Webb A., Smith K., Lee K., Son D.-S., Aschner M., Lee E. (2013). cAMP Response Element-binding Protein (CREB) and Nuclear Factor κB Mediate the Tamoxifen-induced Up-regulation of Glutamate Transporter 1 (GLT-1) in Rat Astrocytes. J. Biol. Chem..

[19] Panikashvili D., Simeonidou C., Ben-Shabat S., Hanuš L., Breuer A., Mechoulam R., Shohami E. (2001). An endogenous cannabinoid (2-AG) is neuroprotective after brain injury. Nature.

[20] Smith M., Wilson R., O’Brien S., Tufarelli C., Anderson S.I., O’Sullivan S.E. (2015). The Effects of the Endocannabinoids Anandamide and 2-Arachidonoylglycerol on Human Osteoblast Proliferation and Differentiation. PLoS ONE.

[21] Basavarajappa B.S., Nagre N.N., Xie S., Subbanna S. (2014). Elevation of endogenous anandamide impairs LTP, learning, and memory through CB1 receptor signaling in mice. Hippocampus.

[22] Panikashvili D., Shein N.A., Mechoulam R., Trembovler V., Kohen R., Alexandrovich A., Shohami E. (2006). The endocannabinoid 2-AG protects the blood–brain barrier after closed head injury and inhibits mRNA expression of proinflammatory cytokines. Neurobiol. Dis..

[23] Eraso-Pichot A., Pouvreau S., Olivera-Pinto A., Gomez-Sotres P., Skupio U., Marsicano G. (2023). Endocannabinoid signaling in astrocytes. Glia.

[24] Siebold L., Obenaus A., Goyal R. (2018). Criteria to define mild, moderate, and severe traumatic brain injury in the mouse controlled cortical impact model. Exp. Neurol..

[25] Cheng H., Wang N., Ma X., Wang P., Dong W., Chen Z., Wu M., Wang Z., Wang L., Guan D. (2022). Spatial-temporal changes of iron deposition and iron metabolism after traumatic brain injury in mice. Front. Mol. Neurosci..

[26] Kraeuter A.K., Guest P.C., Sarnyai Z. (2019). The Open Field Test for Measuring Locomotor Activity and Anxiety-Like Behavior. Methods Mol. Biol..

[27] Lueptow L.M. (2017). Novel Object Recognition Test for the Investigation of Learning and Memory in Mice. J. Vis. Exp..

[28] Kraeuter A.K., Guest P.C., Sarnyai Z. (2019). The Y-Maze for Assessment of Spatial Working and Reference Memory in Mice. Methods Mol. Biol..

[29] Xu X., Jiang S., Xu E., Wu X., Zhao R. (2019). Inhibition of CB1 receptor ameliorates spatial learning and memory impairment in mice with traumatic brain injury. Neurosci. Lett..

[30] Fucich E.A., Stielper Z.F., Cancienne H.L., Edwards S., Gilpin N.W., Molina P.E., Middleton J.W. (2020). Endocannabinoid degradation inhibitors ameliorate neuronal and synaptic alterations following traumatic brain injury. J. Neurophysiol..

[31] Pavlovic D., Pekic S., Stojanovic M., Popovic V. (2019). Traumatic brain injury: Neuropathological, neurocognitive and neurobehavioral sequelae. Pituitary.

[32] Ibáñez I., Díez-Guerra F.J., Giménez C., Zafra F. (2016). Activity dependent internalization of the glutamate transporter GLT-1 mediated by β-arrestin 1 and ubiquitination. Neuropharmacology.

[33] Zhang M., Li W.-B., Liu Y.-X., Liang C.-J., Liu L.-Z., Cui X., Gong J.-X., Gong S.-J., Hu Y.-Y., Xian X.-H. (2011). High expression of GLT-1 in hippocampal CA3 and dentate gyrus subfields contributes to their inherent resistance to ischemia in rats. Neurochem. Int..

[34] Schumm S.N., Gabrieli D., Meaney D.F. (2022). Plasticity impairment exposes CA3 vulnerability in a hippocampal network model of mild traumatic brain injury. Hippocampus.

[35] Hainmueller T., Bartos M. (2020). Dentate gyrus circuits for encoding, retrieval and discrimination of episodic memories. Nat. Rev. Neurosci..

[36] Cui C., Cui Y., Gao J., Sun L., Wang Y., Wang K., Li R., Tian Y., Song S., Cui J. (2014). Neuroprotective effect of ceftriaxone in a rat model of traumatic brain injury. Neurol. Sci..

[37] Sun L., Gao J., Zhao M., Cui J., Li Y., Yang X., Jing X., Wu Z. (2015). A novel cognitive impairment mechanism that astrocytic p-connexin 43 promotes neuronic autophagy via activation of P2X7R and down-regulation of GLT-1 expression in the hippocampus following traumatic brain injury in rats. Behav. Brain Res..

[38] Zhu D., Gao F., Chen C. (2021). Endocannabinoid Metabolism and Traumatic Brain Injury. Cells.

[39] Chen C. (2023). Inhibiting degradation of 2-arachidonoylglycerol as a therapeutic strategy for neurodegenerative diseases. Pharmacol. Ther..

[40] Ahluwalia M., Mcmichael H., Kumar M., Espinosa M.P., Bosomtwi A., Lu Y., Khodadadi H., Jarrahi A., Khan M.B., Hess D.C. (2023). Altered endocannabinoid metabolism compromises the brain-CSF barrier and exacerbates chronic deficits after traumatic brain injury in mice. Exp. Neurol..

[41] Chen X., Zhang J., Chen C. (2011). Endocannabinoid 2-arachidonoylglycerol protects neurons against β-amyloid insults. Neuroscience.

[42] Nomura D.K., Morrison B.E., Blankman J.L., Long J.Z., Kinsey S.G., Marcondes M.C., Ward A.M., Hahn Y.K., Lichtman A.H., Conti B. (2011). Endocannabinoid Hydrolysis Generates Brain Prostaglandins That Promote Neuroinflammation. Science.

[43] Piro J.R., Benjamin D.I., Duerr J.M., Pi Y., Gonzales C., Wood K.M., Schwartz J.W., Nomura D.K., Samad T.A. (2012). A Dysregulated Endocannabinoid-Eicosanoid Network Supports Pathogenesis in a Mouse Model of Alzheimer’s Disease. Cell Rep..

[44] An D., Peigneur S., Hendrickx L.A., Tytgat J. (2020). Targeting Cannabinoid Receptors: Current Status and Prospects of Natural Products. Int. J. Mol. Sci..

[45] Imamura T., Tsuruma K., Inoue Y., Otsuka T., Ohno Y., Ogami S., Yamane S., Shimazawa M., Hara H. (2017). Rimonabant, a selective cannabinoid 1 receptor antagonist, protects against light-induced retinal degeneration in vitro and in vivo. Eur. J. Pharmacol..

[46] Chen Y., Luo X., Liu S., Shen Y. (2018). Neuroprotective effect of cannabinoid receptor 1 antagonist in the MNU-induced retinal degeneration model. Exp. Eye Res..

