# Supplementary material for: Upregulation of GLT-1 Expression Attenuates Neuronal Apoptosis and Cognitive Dysfunction via Inhibiting the CB1-CREB Signaling Pathway in Mice with Traumatic Brain Injury
Source: Biomolecules. 2025 Oct 2;15(10):1408. doi: 10.3390/biom15101408 (PMC12563142; doi:10.3390/biom15101408)
Supplement: Supplementary file 1 [file biomolecules-15-01408-s001.zip › biomolecules-3818247-supplementary.pdf]

Table S1 Antibody for WB and IF

Antibody	Art.No.	Supplier	Dilution ratio	Host	Used for
GLT-1	sc-365634	Santa Cruz	1:500	Mouse	WB
CREB	67927-1-Ig	Proteintech	1:1000	Mouse	WB
P-CREB	ABP0033	Abbkine	1:1000	Rabbit	WB
β-Actin	TA-09	ZSGB-BIO	1:2000	Mouse	WB
GLT-1	sc-365634	Santa Cruz	1:50	Mouse	IF
CREB	sc-377154	Santa Cruz	1:50	Mouse	IF
P-CREB	ABP0033	Abbkine	1:1000	Rabbit	IF
P65	D14E12	CST	1:100	Rabbit	IF
GFAP	60190-1-Ig	Proteintech	1:100	Mouse	IF
GFAP	16825-1-Ig	Proteintech	1:400	Rabbit	IF

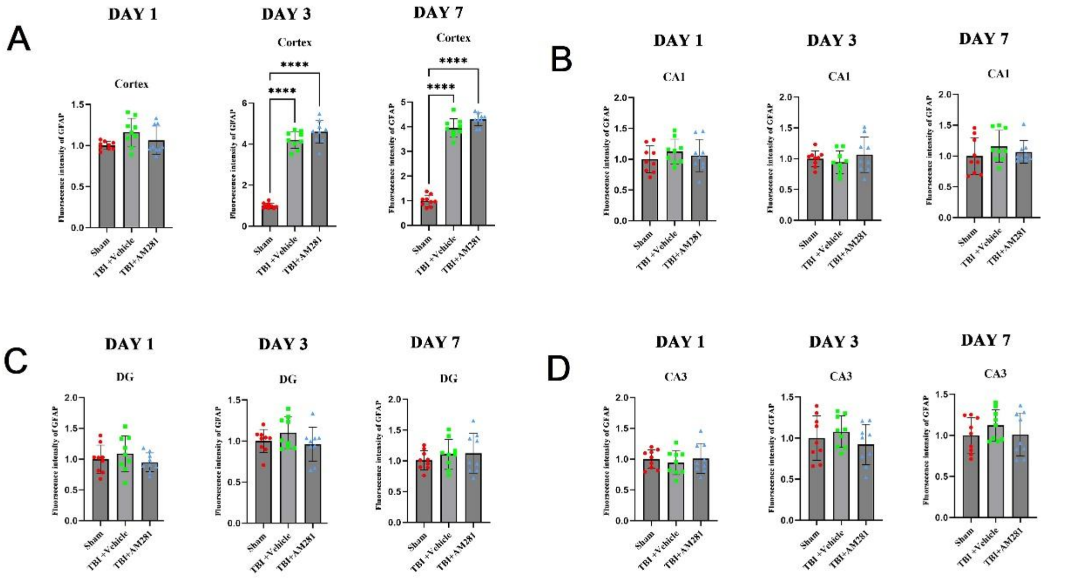

2

Figure S1 TBI-induced upregulation of GFAP expression in astrocytes of cortex was not reversed by inhibiting the CB1 receptor.

A-D: Quantification of immunofluorescence images in Fig.2A-D. n=9 slices from 3 mice per group. All data were presented with the mean ± SEM. ****$p < 0.0001$.

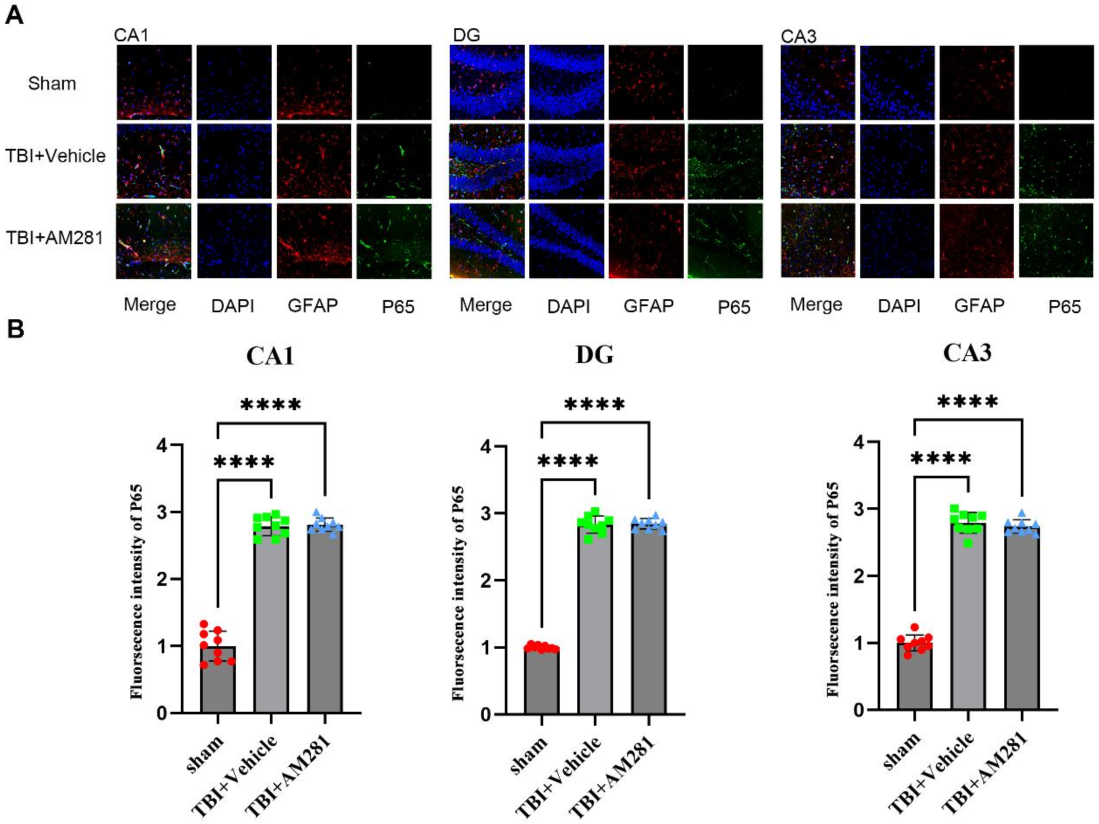

Figure S2 TBI-induced upregulation of P65 expression in astrocytes was not reversed by inhibiting the CB1 receptor.

A: Representative immunofluorescent images demonstrating the expression of GFAP (Red) and P65 (Green) in the CA1, DG, and CA3 of Sham, TBI+AM281, and TBI+Vehicle groups on day 1 post-TBI. B: Quantification of immunofluorescence images in A. n=9 slices from 3 mice per group. All data were presented with the mean ± SEM. ****$p < 0.0001$.
