# Supplementary figures and images for: Upregulation of GLT-1 Expression Attenuates Neuronal Apoptosis and Cognitive Dysfunction via Inhibiting the CB1-CREB Signaling Pathway in Mice with Traumatic Brain Injury
Source: Biomolecules. 2025 Oct 2;15(10):1408. doi: 10.3390/biom15101408 (PMC12563142; doi:10.3390/biom15101408)

figure 1

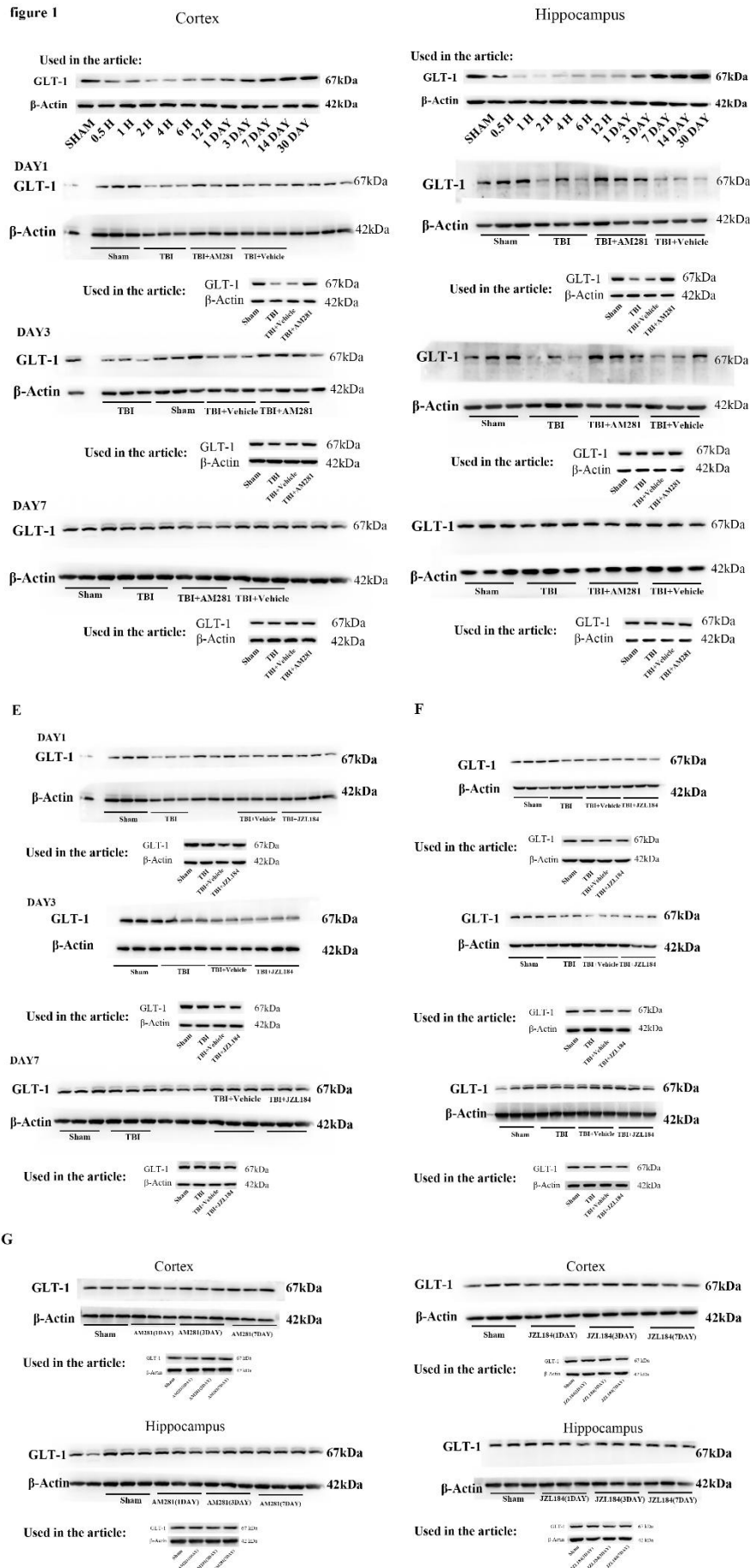

**figure 4-A**

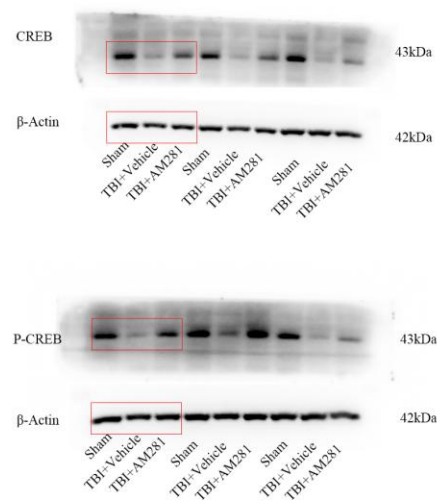

**Used in the article:**

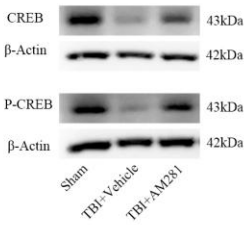

Supplement: Supplementary file 1 [file biomolecules-15-01408-s001.zip › Origianl WB images.pdf]
